# Core, Resident, and Transient Members of the Trout Gut Microbiome: Dietary, Environmental, Stress and Pathogen/Antibiotic Modulation

**DOI:** 10.3390/vetsci13090940

**Published:** 2026-09-10

**Authors:** Albina A. Tsekova, Nadezhda P. Kantserova, Liudmila A. Lysenko, Irina V. Sukhovskaya

**Affiliations:** 1 Institute of Biology, Karelian Research Centre of the Russian Academy of Sciences, 185910 Petrozavodsk, Russia; nkantserova@yandex.ru (N.P.K.); l-lysenko@yandex.ru (L.A.L.); sukhovskaya@inbox.ru (I.V.S.); 2 Institute of Biology, Ecology and Agricultural Technologies, Petrozavodsk State University, 185640 Petrozavodsk, Russia

**Keywords:** *Oncorhynchus mykiss*, rainbow trout, metagenomics, intestinal microbiota, core microbiome, aquaculture

## Abstract

The gut microbiome of rainbow trout plays a crucial role in fish nutrition, immune function and stress resistance, making it a key factor in aquaculture sustainability. This review systematizes metagenomic data that enable the distinction between resident and transient microbial communities and the identification of a core microbiome. At the phylum level, the core is predominantly composed of Bacillota, Pseudomonadota, Actinomycetota, Bacteroidota, and Mycoplasmatota, with additional phyla detected depending on the gut region and methodological approach. Diet-induced changes are often rapid and reversible: insect meal and certain probiotics increase the abundance of beneficial Bacillota and suppress opportunists. Unlike dietary shifts, chronic stressors—particularly elevated temperatures and antibiotic treatment—can override these benefits, causing a persistent alpha diversity reduction, overgrowth of Pseudomonadata, and depletion of key commensals. The autochthonous mucosal microbial community is more resilient to short-term fluctuations but can be irreversibly altered by prolonged stress or antimicrobial therapy, leading to dysbiosis and reduced colonization resistance. A holistic understanding of host–microbe–environment interactions is essential for establishing dynamic reference ranges for a “healthy” microbiome. Such knowledge would enable early detection of dysbiosis and support the rational development of functional feeds and probiotics in commercial trout farming.

## Simple Summary

Gut bacteria are essential for trout health, growth, and immunity, and are therefore critical to sustainable farming. This review shows that while trout harbour a stable core of gut microbes, their composition shifts with diet, stress, and antibiotics. Diet can rapidly and beneficially alter the microbiota, but chronic stress can cause persistent damage, reducing bacterial diversity and weakening disease resistance. Understanding these diet–stress–microbiome interactions is key to designing better functional feeds and probiotics. This knowledge will help detect gut imbalances early and improve trout welfare in commercial aquaculture.

## 1. Introduction

Fish farming is currently one of the fastest-growing sectors of global food production. Among industrial cage aquaculture species, rainbow trout, *Oncorhynchus mykiss* Walbaum, 1792, is one of the most economically significant salmonid species [1]. According to the Food and Agriculture Organization [2], global production is approximately 800,000 tonnes per year. The productivity and well-being of cultured fish are based on the health of their digestive tract.

The fish intestine is a complex, multifunctional organ. Its midsection begins immediately behind the pyloric caeca of the stomach and is characterized by marked differentiation of epithelial, absorptive, and secretory cells. The distal (posterior) part of the salmonid intestine is considered the primary site of macromolecule absorption. As an anatomical continuation of the midgut, it is characterized by a gradual decrease in digestive and transport functions, with a concomitant increase in mucus secretion. In addition to hydrolysis and nutrient sorption, the intestine plays a crucial role in maintaining water and electrolyte balance, endocrine regulation of metabolism, and the development of the immune response. Therefore, maintaining the diversity and functional activity of the intestinal microbiota is critical for fish growth, disease resistance and, ultimately, aquaculture efficiency.

Before examining the taxonomic composition of the trout gut microbiome, it is essential to define the key ecological and operational concepts used throughout this review. In the literature, three related but distinct categories are often conflated: the autochthonous (resident) microbiota, the allochthonous (transient) microbiota, and the core microbiome.

The autochthonous microbiota comprises microorganisms that are evolutionarily adapted to the host, capable of stable colonization, reproduction, and persistence in the gastrointestinal tract. In practice, resident status is inferred from several lines of evidence: (i) consistent detection in the mucosal (parietal) layer across multiple studies; (ii) demonstrated adhesion to mucins or epithelial surfaces; (iii) persistence after dietary or environmental perturbations; and (iv) host-specific co-evolutionary signals. In the rainbow trout literature, the genus *Mycoplasma* is the most consistently validated autochthonous taxon, meeting all these criteria [3,4,5].

The allochthonous microbiota consists of transient microorganisms that enter the gastrointestinal tract from the external environment—primarily via feed or water—and do not form stable, self-sustaining populations. These taxa are typically predominant in the intestinal lumen (chyme), show rapid turnover, and correlate with dietary or environmental inputs. Their abundance declines quickly after removal of the source, distinguishing them from true residents [6,7].

The core microbiome is an operational rather than an ecological category. It is defined as the set of taxa that are consistently detected above a certain frequency threshold (typically >50% of samples) in metagenomic surveys, regardless of geographic origin, age, diet, or rearing conditions [8]. Importantly, the core is a mixed pool: it includes both obligate autochthonous members (e.g., *Mycoplasma*) and stable allochthonous taxa that are regularly introduced from feed or water and persist transiently across multiple sampling time points [3]. Thus, detection frequency alone does not equate to ecological residence.

A critical distinction must be made between spatial biotopes and ecological functions when analyzing metagenomic data. The spatial division of the digestive tract separates the intestinal lumen contents (chyme) from the mucosal (parietal) layer. However, these spatial categories do not perfectly align with the ecological ones defined above. For instance, autochthonous species are constantly displaced into the intestinal lumen during epithelial and mucus shedding, forming a significant proportion of the chyme metagenome. Conversely, allochthonous bacteria from food and water can temporarily accumulate in the parietal mucus without being genuine residents [3]. Distinguishing between these concepts is essential for accurate interpretation of the functional potential of the *O. mykiss* metagenome and for predicting microbiome responses to environmental or therapeutic interventions.

The intestinal microbiota of teleost fish has received considerable attention in recent years due to its fundamental roles in digestion, immunomodulation, and stress resistance. A recent comprehensive review by Tolas et al. [9] provided a valuable meta-analysis of current knowledge in this field, summarizing data across dozens of fish species. However, such broad taxonomic coverage inevitably leads to generalization, which may obscure species-specific features of microbial consortium formation and function. Although studies focused specifically on rainbow trout do exist, most are limited to describing the structure of the basal or so-called core microbiome under normal physiological conditions. Moreover, comprehensive reviews systematizing dynamic shifts in trout microbiota composition and function in response to targeted alterations of external factors are largely lacking.

It should be noted that for the authors of the present work, the topic of the rainbow trout intestinal microbiome is not new. Previously, we published data on the structure of bacterial communities in the trout intestine during *Vibrio* infection [10], and investigated the long-term effects of infection and antibiotic therapy on microbiota composition, as well as muscle and hepatic fatty acids [11]. These studies laid the foundation for understanding stress-induced rearrangements of the microbiome. However, a comprehensive analysis of the dynamics of allochthonous and autochthonous fractions in response to a wide range of external factors has remained inadequately synthesized.

To address this gap, the present study narrows its focus to rainbow trout—a key species in global aquaculture and a model organism for the study of digestive physiology of cold-water fishes. We provide a targeted data synthesis devoted exclusively to the gut microbiome of this species. This approach will enable analysis of the efficacy of strain-specific probiotics and prebiotics developed specifically for trout farming, and to identify specific responses of the trout gut microbiota to stress factors typical of industrial salmon aquaculture (e.g., temperature fluctuations, substitution of fishmeal with plant-based or alternative ingredients, infections).

Accordingly, the aim of this study was to analyze and synthesize data on the composition of the core, allochthonous (transient), and autochthonous (resident) pools of the intestinal microbiota of rainbow trout, as well as their dynamics in response to key environmental factors, stress, and nutrition. Such information enables us to move beyond mere taxonomic enumeration toward an understanding of which microbiome shifts reflect pathological processes (dysbiosis) and which represent physiological adaptation. Furthermore, standardizing approaches for fish health management through microbiome modulation is currently hindered by the absence of a clear reference standard for a “healthy” trout microbiome. Without such a standard, neither early diagnosis of dysbiosis nor the extrapolation of results, for example, from probiotic trials across farms in different geographic regions with varying water quality parameters, is feasible. Finally, a detailed understanding of fish gut microbial dynamics is essential for developing balanced feed formulations that support digestive homeostasis and fish health and welfare.

## 2. Literature Search Strategy

This narrative review synthesizes published literature on the gut microbiota of rainbow trout (*Oncorhynchus mykiss*) in the context of nutrition, environmental factors, antibiotic therapy, and infectious diseases. A comprehensive literature search was conducted in the PubMed electronic database (accessed 17 April 2026). The search strategy employed the following keywords and Boolean operators:

((metagenome[Title/Abstract]) OR (microbiota[Title/Abstract]) OR (microbiome[Title/Abstract]) OR (microbial[Title/Abstract])) AND ((rainbow trout[Title/Abstract]) OR (Oncorhynchus mykiss[Title/Abstract])).

The search covered all publications indexed in PubMed without any time period restrictions.

Inclusion criteria: (i) the study focused exclusively on rainbow trout (*O. mykiss*); (ii) the intestinal microbiota was the sole subject of investigation (microbiomes of other organs or tissues were not considered); (iii) the publication type was an original research article, meta-analysis, or review that described the gut microbiota of trout; (iv) the study assessed the effect of at least one modulating factor; and (v) the article was published in English.

Exclusion criteria: conference abstracts, non-peer-reviewed reports (preprints), studies on the microbiome of other organs and tissues, as well as studies on other fish species without direct comparative data on rainbow trout, were excluded from the review.

Additional relevant publications were identified by manually screening the reference lists of selected articles and key reviews. After completing all selection stages, a total of 77 articles meeting the inclusion criteria were selected for analysis.

## 3. The Core Gut Microbiome of Rainbow Trout

Identifying the core intestinal microbiome is a key area of research in fish physiology. The core microbiome encompasses processes of nutrient absorption, immunomodulation, and the development of colonization resistance—a process by which the normal microbiota outcompetes pathogens for nutrient substrates and mucosal attachment site. The microbial core is defined as a set of taxa that are persistently present in the gastrointestinal tract, regardless of geographic origin, age, diet, season, or rearing conditions.

Dysbiosis of this microbiome serves as an early biomarker of systemic stress and inflammatory processes, and maintaining the integrity of its bacterial community is critical for preserving the barrier function of the epithelium and preventing the translocation of opportunistic microorganisms into the fish bloodstream. A comprehensive inventory of the core bacterial taxa comprising the gut microbiome of rainbow trout, including spatial partitioning between luminal and mucosal communities, is presented in Table 1. Systematic compilation of research results obtained using both classical culture methods and high-throughput sequencing enables identification of a consistent core from the phylum to the species level, and reveals consistent patterns, as well as metagenomic differences influenced by sample type and methodological approach.

Throughout this review, we apply the conceptual definitions of autochthonous, allochthonous, and core microbiota as introduced in the Introduction. To interpret the cited studies, we also specify the operational criteria used to assign taxa to these categories. Autochthonous (resident) status is inferred from mucosal predominance, adhesion capacity, persistence after perturbations, and, in some cases, host-specific phylotypes [3,4,5]. Allochthonous (transient) taxa are characterized by luminal predominance, rapid turnover, and correlation with feed or water inputs [6,7]. The core microbiome is defined operationally by detection frequency thresholds (>50% of samples) and is therefore a mixed pool that includes both residents and stable transients [3,8]. Detection frequency alone does not equate to colonization; complementary approaches (mucosal enrichment, functional metagenomics, culture-based validation) are required to confirm resident status, as demonstrated by recent long-read sequencing studies [12].

The largest meta-analysis, conducted by Hines et al. [8] based on 25 independent studies, showed that regardless of geographic location, fish age, feed type, and the presence of pathogens, the gut microbiome of rainbow trout is consistently composed of representatives of two phyla: Bacillota (formerly Firmicutes) and Pseudomonadota (formerly Proteobacteria). More than 50% of the analyzed studies also identified the phyla Actinomycetota (Actinobacteria), Bacteroidota (Bacteroidetes), and Mycoplasmatota (Tenericutes). Family- and genus-level analysis revealed clear spatial differentiation of microorganisms within the gastrointestinal tract. Within the phylum Bacillota, the family Lactobacillaceae was identified predominantly in the allochthonous (transient) microbiota pool, while Streptococcaceae and Clostridiaceae were mainly found in the intestinal lumen. Within the phylum Pseudomonadota, the most common members were Enterobacteriaceae, Pseudomonadaceae, and Moraxellaceae (including the genus *Acinetobacter*). Among Bacteroidota, the family Flavobacteriaceae dominated, with its members being significantly enriched in the mucosal layer, indicating their strong adhesion properties. Notably, this family also includes pathogens dangerous to salmon farming, *Flavobacterium columnare* and *F. psychrophilum*, whose constant presence in the core microbiome confirms their status as opportunistic residents. Within the phylum Mycoplasmatota, the family Mycoplasmataceae (genus *Mycoplasma*) predominated. This taxon was detected in the intestinal mucosa substantially more frequently than in the intestinal lumen, confirming its strictly autochthonous nature, and under specific conditions (e.g., stress or dysbiosis), it became a major component of the entire microbial community.

The taxonomic structure of the trout intestinal core microbiome is strictly determined by the anatomical and physiological heterogeneity of the gastrointestinal tract, forming two distinct biotopes: the chyme and the mucosal layer. The intestinal luminal contents represent an open, dynamic system with high alpha diversity, dominated by allochthonous (transient) microbial populations. Bacteria from the families Aeromonadaceae (primarily *Aeromonas salmonicida*, *A. media*, *A. sobria*), Enterobacteriaceae (genera *Proteus*, *Enterobacter*, *Buttiauxella*, *Plesiomonas*, *Hafnia*, *Pantoea*), and Pseudomonadaceae predominate in this niche [3]. The high density of opportunistic Enterobacteriaceae and Aeromonadaceae in the chyme reflects their constant intake via feed or water. In addition, high proportions of the phyla Bacteroidota and Fusobacteriota are regularly detected in the chyme; these taxa are generally unable to attach to host tissues and are eliminated along with undigested food residues [3].

In contrast, the parietal mucus layer functions as a highly specialized filter. It is predominantly home to an autochthonous community characterized by lower taxonomic diversity but high compositional stability, regardless of rearing conditions [4,5]. The predominant taxon of the mucosal layer is the family Mycoplasmataceae, with the genus *Mycoplasma* (including *Candidatus Mycoplasma salmoninae mykiss*), the relative abundance of which in the parietal mucus of healthy individuals can reach 68–81% [3,4]. High abundance also characterizes the phylum Pseudomonadota (primarily the class Gammaproteobacteria), where the major OTUs were the genera *Psychrobacter*, *Acinetobacter*, *Pseudomonas*, and *Cetobacterium*. Minor but persistent core components included the genera *Aeromonas*, *Clostridium*, *Deefgea*, *Flavobacterium* and *Neptunibacter* [5]. Resident mucosal taxa possess a selective advantage: they exhibit high adhesion to mucins and biosynthesize antibacterial metabolites, which block epithelial colonization through both physical and chemical mechanisms.

Similar patterns of core microbiome stability were reported by Lyons et al. [4] comparing trout from commercial cage culture and controlled aquarium conditions. In both groups, the phyla Mycoplasmatota, Bacillota, Pseudomonadota, Spirochaetota, and Bacteroidota dominated. At the same time, the genus *Mycoplasma* predominated within Mycoplasmatota. The phylum Bacillota was represented predominantly by the classes Bacilli (genera *Lactobacillus*, *Catellicoccus*, *Streptococcus*, *Weissella*, *Leuconostoc*, *Lactococcus*, *Enterococcus*, *Bacillus*) and Clostridia (genus *Acetanaerobacterium*). The profile of Pseudomonadota was characterized by the class Gammaproteobacteria, with high abundance of the genera *Photobacterium*, *Pseudomonas*, *Acinetobacter*, *Maricurvus*, *Moritella*, *Pantoea*, along with minor representation of Alphaproteobacteria and Betaproteobacteria.

Historically, identification of the *O. mykiss* core microbiota was limited to the genus level due to the methodological limitations of conventional short-read NGS. With the development of long-read amplicon sequencing technologies for the full-length 16S rRNA gene, it has become possible to define the core microbiome of the trout gut at the species level, with identified species collectively comprising up to 91% of the entire gut bacterial community [12]. The species profile of the autochthonous microbiome pool reported to date includes *Pseudomonas yamanorum*, *Malacoplasma muris*, *Latilactobacillus sakei*, *Ralstonia pickettii*, *Rhodococcus qingshengii*, *Deefgea piscis*, *Aeromonas sobria*. The expanded list of identified resident microorganisms includes *Lactobacillus fuchuensis*, *Mesomycoplasma moatsii*, *Clostridium gasigenes*, *Cetobacterium somerae*, *Weissella koreensis*, *Deefgea salmonis*, *Lactococcus cremoris*, *Carnobacterium maltaromaticum*, *Leuconostoc gasicomitatum*, *Shigella sonnei*, *Escherichia coli*, *Bacillus nitratireducens*, and several species of the genus *Ralstonia* (*R. solanacearum*, *R. insidiosa*, *R. nicotianae*). The presence of *Candidatus Mycoplasma salmoninae mykiss* was confirmed with high sequence identity. The use of a higher-resolution method allowed reassessment of the resident status of several genera previously considered part of the trout core microbiome. *Streptococcus*, *Methylobacterium*, *Staphylococcus*, *Shewanella*, and *Corynebacterium* were not detected by full-length sequencing [12]. These findings highlight those methodological factors, such as primer selection, amplicon length, and DNA extraction protocols, play a crucial role in accurately characterizing the trout gut metagenome.

The studies reviewed employ diverse methods—culture-dependent approaches, short-read 16S rRNA sequencing, and full-length 16S rRNA sequencing—each with inherent biases. Culture-dependent methods recover only a small fraction of microbial diversity due to the unculturability of most gut bacteria [8]. Short-read sequencing offers high throughput but limited taxonomic resolution, often restricting identification to genus level, with primer choice introducing significant amplification bias [8,12]. Full-length sequencing substantially improves species-level resolution but is more costly, has lower throughput, and its accuracy depends on reference database completeness [12]. These methodological differences may explain discrepancies in core composition across studies: for example, the presence of *Streptococcus*, *Staphylococcus*, and *Corynebacterium* in some short-read surveys [4,8] and their absence in full-length studies [12] likely reflects improved resolution rather than true biological variability. Thus, cross-study comparisons must consider methodological context.

In summary, the rainbow trout gut microbiome exhibits a stable phylogenetic framework. Its core, which remains stable across diverse environmental conditions, is formed by representatives of the phyla Mycoplasmatota, Pseudomonadota, and Bacillota, along with Fusobacteriota, Spirochaetota, and Bacteroidota. Furthermore, within the digestive tract, a strict spatial organization is observed, dividing the microbiota into two functionally distinct biotopes. The intestinal lumen is characterized by high taxonomic diversity, temporal variability, and the dominance of allochthonous species, including members of the Enterobacteriaceae and opportunistic Aeromonadaceae families. In contrast, the parietal mucus layer represents a highly specialized niche with reduced alpha diversity, where the obligate autochthonous genus Mycoplasma dominates, along with the mucosa-associated genera *Psychrobacter*, *Acinetobacter* and *Pseudomonas*. Maintaining the stability of this parietal pool, which includes the resident species *Pseudomonas yamanorum*, *Malacoplasma muris*, and *Latilactobacillus sakei*, is critically important for host health, epithelial barrier integrity, and colonization resistance. Notably, the recent transition from short-read analysis to long-read sequencing of the full-length 16S rRNA gene has demonstrated that the detection of several taxa (e.g., *Streptococcus*, *Methylobacterium*, and *Staphylococcus*) previously considered part of the core microbiome is highly method-dependent. Thus, the identified species diversity holds promise for the development of probiotic preparations and diagnostic markers, but requires further validation under controlled experimental conditions, with particular attention to the methodological approach and sampling site.

## 4. Factors Modulating the Rainbow Trout Gut Microbiome

The intestinal microbiome of fish is a dynamic ecosystem closely linked to the host organism. It plays a key role in digestion, immune regulation, and the maintenance of colonization resistance. The allochthonous and autochthonous microbial fractions exhibit markedly different lability. The allochthonous (transient) pool acts as a highly sensitive marker, responding rapidly to changes in external conditions. Its structure is directly influenced by diet, temperature, and water quality. In contrast, the autochthonous mucosal community exhibits high resistance and stability, ensuring the integrity of the mucosal barrier. However, resident microorganisms are also susceptible to perturbations: chronic or severe stressors can overcome the buffering capacity of the mucus layer and cause profound and sometimes irreversible changes in the taxonomic composition of the microbiome. This section reviews the major groups of modulatory factors, including dietary, abiotic, and infectious agents, and their impact on the trout gut microbiota. A summary of the effects of dietary interventions, rearing systems, stress, and pathogen/antibiotic exposure on the gut microbial community is presented in Table 2.

### 4.1. Dietary Modulation of the Rainbow Trout Intestinal Microbiota

Diet is a major exogenous factor shaping the gut microbial community composition of fish. The composition and diversity of the microbial community vary significantly depending on nutrient sources (proteins, lipids, and carbohydrates) and the inclusion of functional feed additives. High-throughput metagenomic approaches enable detailed characterization of the taxonomic composition and the identification of specific changes associated with the replacement of fishmeal with plant-based or animal-based alternatives. Understanding how dietary factors modulate the gut microbiome offers opportunities for optimizing feed formulations and improving the health of rainbow trout in aquaculture.

Current research on diet–microbiome interactions in rainbow trout focuses on several key areas. The largest body of research addresses the replacement of traditional fishmeal with alternative protein sources, including plant-based ingredients (peas, soybean, rapeseed, lupine, wheat), insect meals (*Hermetia illucens* larvae, *Tenebrio molitor* mealworms, *Gryllodes sigillatus* crickets, and *Zophobas morio*), as well as poultry by-products, yeast (*Saccharomyces cerevisiae*) and various combinations thereof.

Replacing fishmeal with plant-based ingredients results in compositional restructuring of the community and an increase in the Bacillota-to-Pseudomonadotaratio [13,14,15]. A plant-based diet rich in carbohydrates and fiber shifts the microbiota toward dominance of lactic acid bacteria of the genera *Streptococcus*, *Leuconostoc*, and *Weissella*, whereas a traditional fishmeal-based diet maintains the predominance of Pseudomonadota [16,17]. The severity of dysbiosis correlates with the degree of raw material processing: the use of highly purified protein concentrates minimizes risks compared to unrefined plant meal, as concentrates do not induce mucosal inflammation and help maintain the stability of the autochthonous community [13].

The inclusion of insect meal in feed is considered one of the most promising strategies. In most studies, the use of *H. illucens* larvae was associated with a pronounced prebiotic effect: an increase in the proportion of Bacillota, a decrease in Pseudomonadotalevels, an increase in the abundance of beneficial taxa (*Lactobacillus*, *Bacillus*, *Carnobacterium*, *Oceanobacillus*, *Paenibacillus*), and suppression of the potentially pathogenic genus *Aeromonas* [18,19,20,21]. Chitin appears to be the key factor, serving as a substrate for chitinolytic bacteria and stimulating short-chain fatty acid production. Findings for other insect species have been more variable: complete replacement of fishmeal with *T. molitor* meal did not induce adverse effects, but rather resulted in moderate modulation [22,23], whereas diets containing the cricket *Gryllodes sigillatus* reduced alpha diversity of the microbiota [21]. A key conclusion of these studies is the high plasticity of the rainbow trout microbiota and its ability to rapidly and reversibly adapt to the current diet, without long-term imprinting by starter feeds [17,72,73]. However, short-term exposure to highly specialized diets during the first feeding period can cause persistent, long-term changes in the mycobiota, whereas the bacteriobiota of adult fish returns to baseline after a dietary shift [74].

A second line of research focuses on the use of probiotics, prebiotics, postbiotics, and synbiotics. *Pediococcus acidilactici*-based probiotics have shown variable effects, with some studies reporting minimal effects on overall microbiota structure, whereas others have demonstrated effective stabilization during *Yersinia ruckeri* infection, significantly reducing dysbiosis [18,24,25]. In contrast, multispecies probiotics alter the profiles in a dose-dependent manner, increasing gut microbial biodiversity [26]. Targeted microbiome restructuring—characterized by an increased abundance of beneficial bacteria (including representatives of the Lachnospiraceae family, the genus *Ruminococcus*, and *Bacillus coagulans*) alongside a reduction in pathogenic taxa—has been observed following the administration of *Bacillus velezensis* and *Lactobacillus sakei* [27]. *Carnobacterium maltaromaticum* demonstrates pronounced antagonism towards opportunistic bacteria, reducing their numbers by 2–6 orders of magnitude [28]. The use of *Bacillus cereus* var. *Toyoi* leads to homogenization and restructuring of the microbiota, resulting in a more stable composition that is less variable among individuals [29], whereas *Weissella confusa* exerts a pronounced stimulatory effect directly on the autochthonous lactic acid bacterial population [30]. Prebiotic supplementation also enables selective modulation of the trout gut microbiota. Mannan oligosaccharides reduce the bacterial load and the presence of opportunistic genera *Aeromonas* and *Vibrio*, while increasing overall diversity and the relative abundance of Bacillota (Firmicutes) and Fusobacteria [75,76]. Dietary inulin exerts a more selective effect: it modulates the chyme microbiota but does not affect the parietal microbiota, increasing the abundance of *Weissella* and *Streptococcus* [31], although in other studies its effect was minimal or even detrimental to growth performance [32]. β-glucan from *Saccharomyces cerevisiae* dose-dependently increases the proportion of Actinobacteria, particularly the genus *Aurantimicrobium*, but reduces the relative abundance of Bacillota, including the genera *Carnobacterium* and *Deefgea* [33]. The inclusion of arabinoxylan in the diet at high concentrations (10%) suppresses alpha diversity, increases the Firmicutes-to-Bacteroidetes ratio, and promotes the growth of opportunistic *Stenotrophomonas* bacteria [34]. Similarly, soluble non-starch polysaccharides disrupt intestinal homeostasis by increasing chyme viscosity, leading to an increase in the proportion of potentially pathogenic bacteria, including Pseudomonas aeruginosa and *Photobacterium kishitanii* [35]. In contrast, postbiotics have been recognized as an effective tool for optimizing digestion, as their administration increases the alpha diversity of the intestinal microbiota, elevates the Tenericutes-to-Fusobacteria ratio, and suppresses sulfate-reducing bacteria of the genus *Desulfovibrio* [36,37].

The third group of dietary factors consists of organic acids, essential oils, and plant extracts. Mixtures of organic acids and essential oils have a moderate modulatory effect: they reduce the abundance of *Aeromonas hydrophila* and *Acinetobacter*, while increasing the proportion of *Streptococcus* and *Fusobacterium*, without inducing major community-level shifts [42,43,44]. However, at elevated temperatures, these mixtures do not prevent a decline in overall biodiversity and a reduction in the abundance of *Leuconostoc* and *Streptococcus*. Sodium butyrate supplementation increases species richness and alters microbiome structure. Specifically, when administered in combination with a high-fish diet, Firmicutes are replaced by Proteobacteria, and the dominant genus shifts from *Mycoplasma* to *Aeromonas* [45,46]. Moreover, sodium butyrate at a dose of 0.20% reduces the abundance of opportunistic Proteobacteria [46]. Capsaicin (derived from *Capsicum* spp.) increases alpha diversity and the abundance of Clostridiaceae, primarily affecting rare taxa [38,39]. Plant extracts (garlic, Chinese yam, and *Ganoderma lucidum*) and mushroom-derived polysaccharides (lentinan from shiitake) effectively restore microbial homeostasis following viral infections, particularly those caused by infectious hematopoietic necrosis virus (IHNV), by suppressing the pathogenic genera *Mycobacterium* and *Nannocystis* [38,39,40]. Mushroom stipes of *Agaricusbisporus*, *Lentinulaedodes*, and *Pleurotusostreatus* increase alpha diversity and induce site-specific shifts along the gastrointestinal tract, reducing the proportion of Desulfobacterota and *Staphylococcus* while increasing beneficial taxa [41]. Micronutrients also influence the microbiota: copper(I) complexes promote the growth of *Pseudomonas* and *Corynebacterium* [47], while glutathione (400 mg/kg) enhances alpha diversity and inhibits the genus *Arcobacter* [48].

The combined effect of nano-selenium (nano-Se) and acute heat stress provides a well-documented example [49]. Acute heat stress (24 °C) causes a sharp depletion of microbial richness, with the number of unique OTUs decreasing from 237 to 73, and an abnormal increase in the Simpson index, driven by overgrowth of opportunistic taxa. At the phylum level, under elevated water temperatures, the relative abundance of the core genus *Ralstonia* and the phylum Pseudomonadota decreases by approximately 25%, giving way to Actinomycetota and Bacillota, whereas *Methylobacterium*, *Akkermansia*, and *Deinococcus* emerge as stress-associated biomarkers. These results show that dietary supplementation with nano-selenium (5 mg/kg) mitigates the described dysbiotic shifts, restoring alpha- and beta-diversity indices and returning dominant phylum profiles to baseline levels observed at 18 °C.

A further example of long-term adaptation and resilience of the microbial community to combined dietary and stocking density factors is provided by a large-scale 214-day study conducted by Wong et al. [50], in which the authors assessed the combined effects of dietary transition from fishmeal to plant-based ingredients and of stocking density (high vs. low) on adult *O. mykiss*. The experiment demonstrated that despite significant changes in fish growth performance, the intestinal microbiota maintained homeostasis. Regardless of the combination of factors, all trout groups exhibited a stable phylogenetic core represented by the classes Bacilli, Clostridia, Alphaproteobacteria, Gammaproteobacteria, and Betaproteobacteria. Histological analysis revealed no signs of inflammation or mucosal destruction. External factors induced only moderate shifts in a minor fraction of the microbiome: a grain-based diet combined with low stocking density enhanced the diversity of *Bacillus* spp., whereas fishmeal combined with high stocking density increased the abundance of *Clostridium*. The use of plant-based ingredients also selectively increased the abundance of potentially probiotic genera *Lactobacillus* and *Streptococcus*, with a significant synergistic effect of these two factors observed exclusively for the genus *Staphylococcus*.

In summary, dietary modulation of the rainbow trout gut microbiome is highly effective but context-dependent. Alternative protein sources (insect meal, plant proteins) and certain probiotics consistently increase Bacillota and suppress opportunistic Pseudomonadota, while prebiotics and postbiotics show more variable effects. However, the magnitude and direction of response depend on dosage, basal diet composition, and environmental conditions. Key knowledge gaps include the long-term persistence of diet-induced shifts after dietary restoration and the mechanisms underlying strain-specific probiotic effects.

### 4.2. Rearing System and Biotope Effects on the Trout Gut Microbiome

Housing conditions of rearing systems designs are among the key factors determining the composition and function of microbial communities in the gastrointestinal tract of rainbow trout. In their natural habitat, the gut microbiome is shaped by seasonal succession, hydrological fluctuations, and a highly heterogeneous food supply. In contrast, in intensive aquaculture, farmed fish are isolated from natural cycles and exposed to continuous pressure from anthropogenic factors. The transition from wild populations to cage culture, as well as the choice between flow-through systems and recirculating aquaculture systems (RAS), can alter the metagenomic profile of the trout gut microbiome. This section provides an overview of studies examining the influence of different aquatic environments and rearing systems on the taxonomic structure of the trout gut microbiota. Understanding these relationships is critical for optimizing health, immune status, and feed conversion efficiency in fish farming.

Comparative analysis of the gut metagenome of wild trout and aquaculture specimens reveals fundamental differences in the structure of the core microbiome. A typical example is provided by profiling data from wild rainbow trout from a remote Alaskan river system, where the native microbiome baseline was established [51]. Under natural conditions, the dominant phyla in the gut contents are Bacillota and Fusobacteriota, followed by Cyanobacteria, Pseudomonadota, and Bacteroidota. At the genus level, *Cetobacterium* and *Clostridium sensu stricto* are predominant. Notably, the intestines of wild populations entirely lacked genera such as *Mycoplasma*, *Pseudomonas*, and *Weissella*, which are traditionally dominant and considered core for farmed trout. This is confirmed by comparative studies documenting profound changes in the taxonomic profile even after trout were transferred from open-air lake farms to controlled laboratory aquaria [4]. Commercially farmed fish exhibited higher prevalence of genera *Photobacterium*, *Catellicoccus*, *Moritella*, *Ureibacillus*, *Paralactobacillus*, *Psychrilyobacter*, *Thermobacillus*, *Lactobacillus*, and *Fusobacterium*, whereas aquarium-reared fish were enriched in the genera *Sphaerotilus*, *Maricurvus*, and *Weissella*. Flowing lake water induces the formation of a community dominated by anaerobic and aerotolerant representatives of Bacillota and Bacteroidota, whereas closed aquaculture systems result in a marked shift toward specialized genera adapted to biofilters and plastic surfaces.

Assessment of the combined effects of diet type and rearing system revealed that the rearing system type (RAS or flow-through) exerts a significantly stronger modulatory effect on the gastrointestinal microbiome than dietary variations [52]. Across all samples examined, the dominant bacterial phyla were Pseudomonadota, Bacillota and Bacteroidota. The aquatic environment is the primary source of intestinal colonization by facultative anaerobic genera *Aeromonas* and *Acinetobacter*. The genera *Enterobacter*, *Lactococcus*, *Paracoccus*, and *Succinipira* predominated in the intestinal lumen, while the mucosa was enriched in *Tolumonas*, *Chitinophaga*, *Enhydrobacter*, *Alcaligenes*, *Arcobacter*, *Brevundimonas*, and representatives of the suborder Corynebacterineae (within the phylum Actinomycetota), aa well as the genus *Clostridium*. In commercial flow-through farms, anaerobic or aerotolerant fermenting genera *Lactobacillus*, *Lactococcus*, *Clostridium*, *Catellicoccus*, *Fusobacterium*, *Ureibacillus*, *Paralactobacillus*, and *Thermobacillus* naturally accumulate in the trout gut [4,52]. In contrast, in RAS and laboratory aquaria, the proportion of the class Gammaproteobacteria (genera *Aeromonas*, *Lelliottia*, *Maricurvus*) and certain genera of Bacillota (*Weissella*, *Enterococcus*, *Streptococcus*) tends to increase, with *Mycoplasma* often predominating in the anaerobic niche of the distal intestine [4,52,53].

Despite considerable influence from the environmental microbiome, the healthy trout intestine functions as a strictly isolated biofilter. Analysis of the hindgut in RAS conditions showed that it is strongly isolated from other mucosal organs (gills, oropharynx, skin) and the microbial environment of recirculating water and biofilms [53]. The intestinal microbiota is characterized by the lowest alpha diversity and estimated richness compared to all other tissues. While Pseudomonadota dominate the gills, skin, and water, strict anaerobiosis creates a unique environment in the distal intestine, where *Mycoplasma* can accumulate up to 66.7% of all metagenomic reads. Strict aquatic aerobes (such as *Flavobacterium* and *Crocinitomix*) are largely excluded from the gastrointestinal tract, being replaced by facultative and obligate anaerobes. However, this autonomy varies considerably among individuals and between farms. For example, in Danish trout farms, the gut microbiome may be dominated either by Enterobacteriaceae or by a mixture of *Carnobacterium*, *Pseudomonas*, *Shewanella*, *Acinetobacter*, and *Plesiomonas*, depending on the specific farm and season [54]. The analysis reveals the dominance of specific resident taxa, such as *Carnobacterium piscicola*, *Clostridium botulinum*, and uncultured coccoid bacteria that exhibit only 89% similarity to the well-known genus *Anaerofilum*.

Overall, the rearing environment exerts a strong influence on the trout gut microbiome, often exceeding the effect of dietary interventions. Wild fish harbor distinct communities dominated by Bacillota and Fusobacteriota, whereas farmed and aquarium-reared fish show enrichment in Pseudomonadota and Mycoplasmatota. The gut microbiome exhibits autonomy from other mucosal sites, maintaining a unique anaerobic community. However, substantial variation exists between farms and seasons, and the long-term consequences of microbiome shifts induced by RAS versus flow-through systems remain poorly understood.

### 4.3. Stress-Induced Plasticity and Resistance of the Rainbow Trout Gut Microbiota

Stressors in aquaculture trigger homeostasis disruption through multiple pathways. Physiological responses to stress induce changes in the gastrointestinal tract, including epithelial barrier damage, alterations in mucus secretion and dysbiosis. Recent high-throughput sequencing demonstrates that both acute and chronic stress cause profound changes in the taxonomic composition of the autochthonous and allochthonous microbiota, often accompanied by a decrease in alpha diversity and an increase in the proportion of opportunistic taxa. This section presents an analysis of studies assessing structural shifts in trout intestinal bacterial communities under various stress factors.

Acute stress, induced by a rapid drop in water level and brief mechanical pursuit, induced structural shifts in the intestinal microbiota of trout [55]. Analysis revealed a two-order-of-magnitude reduction in the abundance of culturable aerobic bacteria in the mucus layer of the distal intestine of satiated fish within 4–48 h post-stress. This process occurred concurrently with an equivalent increase in the density of the same bacteria in feces, supporting the hypothesis of stress-induced shedding of the intestinal mucus gel. Qualitative shifts included the disappearance of *Acinetobacter* and *Rhodococcus* from the parietal community and the establishment of *Pseudomonas* dominance; *Arthrobacter*, *Microbacterium*, and *Micrococcus*, which had not previously been detected in these samples, appeared in the fecal microbiota after stress. In individuals fasted for three days, the buffering capacity of the parietal mucus appeared more resistant to acute stress, suggesting a physiological relationship between gastrointestinal chyme load and the severity of stress-induced epithelial detachment.

Prolonged (chronic) stress induced by repeated handling (e.g., systematic chasing with a net) has a long-term modulating effect, the nature of which is determined by host genotype and diet [56,57]. Chronic stress can differentially alter alpha and beta diversity indices in the intestinal lumen of trout [56]. In selectively bred trout strains with inherently high stress susceptibility, prolonged stress exposure causes an increase in alpha diversity in the intestinal contents regardless of diet type, whereas in resistant strains, this index remains stable. At the same time, beta diversity in stress-susceptible strains is strongly influenced by stress, whereas in resistant fish, a complex synergistic interaction between stress and diet is observed. At the phylum level, the primary marker of chronic stress in the intestinal contents of trout is an increase in the abundance of Fusobacteriota and its key representative, the genus *Cetobacterium* [56]. It has also been shown that the combined effect of stress and a fishmeal-based diet leads to a marked enrichment of the chyme with *Cetobacterium*, *Photobacterium* and *Plesiomonas*, whereas a plant-based diet increases the abundance of *Bifidobacterium* and *Candidatus Microthrix*. Comparative analysis of the mucosal and luminal microbiomes under chronic stress reveals spatial partitioning [57]. While the intestinal contents are characterized by a higher number of amplicon sequence variants than the mucosa, and the dominant taxa are Bifidobacterium, Staphylococcus, Corynebacterium, and Bacteroides, stress influences the mucosal microbiota primarily by modulating dietary effects. The intestinal mucosa of stressed fish is dominated by *Mycoplasma*, *Cetobacterium*, *Photobacterium* and *Brevinema*, with *Mycoplasma* remaining the major commensal.

Optimizing stocking density in recirculating water systems is a key factor in maintaining microbial homeostasis. Contrary to common assumptions, chronic stress and profound dysbiosis can be induced not only by extremely high but also by excessively low stocking densities (as low as 12 kg/m^3^) [58]. Insufficient stocking density triggers behavioral stress (suppression of schooling behavior), leading to an increase in the abundance of opportunistic pathogens *Pseudomonas putida*, *Acinetobacter lwoffii*, and *Pseudomonas alcaligenes* and members of the genus *Shewanella*. In contrast, a moderate, technologically appropriate stocking density (approximately 43 kg/m^3^) mitigates these shifts by promoting the accumulation of immunomodulatory commensals, *Cetobacterium somerae*, *Romboutsia lituseburensis* and *Lactobacillus plantarum*.

A similar disruptive pattern of transient dysbiosis is induced by forced starvation or severe feed restriction [59]. Food deprivation triggers a marked decline in *Bifidobacterium* abundance, accompanied by a concurrent increase in opportunistic genera including *Helicobacter*, *Staphylococcus* and *Pseudomonas*. This shift is reversible: resumption of standard feeding restores the native structure of both autochthonous and allochthonous microbial pools within 7–14 days.

Water temperature, a major abiotic stressor for stenothermic rainbow trout, can override or exacerbate dietary effects [60]. Increasing water temperature to the upper thermal optimum (18 °C) leads to a significant decrease in bacterial alpha diversity, which is most pronounced with standard fishmeal-based diets. The addition of live yeast *Saccharomyces cerevisiae* to the feed exerts a partial protective effect, maintaining bacterial diversity at a higher level. Temperature regime affects the composition of dominant taxa: Mycoplasmatales are abundant under a yeast-supplemented diet at 18 °C, whereas Lactobacillales predominate at 11 °C with a standard diet [60]. Cold water combined with fishmeal stimulates the accumulation of lactic acid bacteria, including Leuconostocaceae, *Lactobacillus reuteri*, and the genus *Photobacterium*, whereas warming to 18 °C shifts dominance towards the opportunistic family Aeromonaceae in the chyme, while the parietal microbiome remains relatively stable [60].

Moreover, temperature shift exerts a dominant influence on microbial community structure, exceeding the effect of substituting animal protein for plant protein (soy, lupine, wheat) [61]. Regardless of the protein and fat source, a thermal regime of 14 °C maintains the dominance of autochthonous Mycoplasmataceae, whereas a water temperature of 18 °C triggers a restructuring with the replacement of the core by opportunistic families Aeromonadaceae and Enterobacteriaceae, which act as key taxa indicator of heat stress [61].

Acute temperature stress (exposure to temperatures of 22.5–24.5 °C for 24 h) provokes a sharp drop in alpha diversity and a restructuring of beta diversity [35]. During extreme short-term overheating, an increase in the relative abundance of Mycoplasmatota and Bacillota was recorded at the phylum level. Within these groups, strong increases were observed in the genera *Mycoplasma*, *Cetobacterium*, *Aeromonas*, *Shewanella*, and *Clostridium*, while the abundance of *Lactobacillus* spp. and the genera *Coldibacterium*, *Morganella*, *Enterobacter*, and *Lawsonia* decreased. LEfSe analysis identified markers associated with specific temperature regimes. In the control group (16 °C), these included *Cloacibacterium* (in particular, *C. normanense*), the Prevotellaceae family, and the genera *Microbacterium*, *Morganella*, and *Lactobacillus fermentum*. For the “22.5 °C” group, the biomarkers were representatives of *Mycoplasma* (including *Mycoplasma moatsii* and *Mycoplasma penetrans*), the Mycoplasmataceae family, and the phylum Tenericutes. At 23.5 °C, the Firmicutes phylum (especially the class Bacilli) emerged as a marker, while at 24.5 °C, the class Betaproteobacteria emerged as a marker. The authors conclude that even short-term (24 h) heat stress significantly restructures the gut community, reducing its diversity and promoting the growth of potentially pathogenic taxa (*Aeromonas*, *Shewanella*) and *Mycoplasma* while suppressing beneficial Lactobacilli and *Cloacibacterium*.

Prolonged heat stress (24 °C for 21 days) disrupts the mucosal barrier [62,63]. Under chronic hyperthermia, the total proportion of Bacillota and Bacteroidota decreases, while Pseudomonadota increases throughout the gastrointestinal tract. Alpha diversity indices may not change, but the Chao1 index in intestinal contents significantly increases, indicating stochastic accumulation of opportunistic taxa from the surrounding environment [62,63]. In the mucosal niche, chronic temperature stress reduces the abundance of the obligate autochthonous genus *Mycoplasma* (including *Mycoplasma suidaniae* and *Mycoplasma agassizii*-like OTUs). These are replaced by uncultured representatives of Enterobacteriaceae (genera *Escherichia-Shigella* and *Klebsiella*) and *Aeromonas* (*Aeromonas veronii*, *Aeromonas hydrophila*) [63]. In the intestinal contents, chronic temperature stress suppresses populations of *Bacillus*, *Clostridium* (*C. butyricum*), and *Acinetobacter* (*A. johnsonii*, *A. lwoffii*). These are replaced by uncultured representatives of the tribe Escherichieae [62]. This profound dysbiosis correlates with systemic disturbances in lipid and choline metabolism. These disturbances lead to enteritis, damage to the intestinal epithelial barrier, and translocation of bacterial pathogens into the bloodstream.

In summary, stress, particularly elevated temperature, triggers consistent and often profound shifts in the trout gut microbiome, characterized by decreased alpha diversity, overgrowth of Pseudomonadota (Enterobacteriaceae and *Aeromonas*), and depletion of commensal Bacillota and *Mycoplasma*. These effects can override beneficial dietary interventions and compromise mucosal barrier function. Chronic stress causes more persistent alterations than acute stress, and recovery is incomplete under continued exposure. Important gaps remain regarding the threshold temperatures for irreversible dysbiosis and the efficacy of mitigation strategies (e.g., probiotics, feed additives) under chronic stress conditions.

### 4.4. Pathogen-Associated and Antibiotic-Induced Microbiota Shifts in Rainbow Trout

Infectious and mycotic diseases, particularly in cage farming systems, trigger dysbiosis through host immune responses and altered physicochemical conditions [77]. This process results in a decrease in alpha diversity, depletion of obligate commensals, and uncontrolled expansion of opportunistic microbiota. High-resolution metagenomic analysis enables the identification of key patterns of these shifts depending on spatial localization, thermal conditions, infection stage, and subsequent therapeutic load.

Antibiotics, typically administered orally via feed or as therapeutic baths, pose substantial ecological risks, primarily through the selection of resistant bacterial strains and the dissemination of antibiotic resistance genes in aquatic ecosystems. Consequently, research has shifted toward investigating the long-term effects of antibiotic therapy on the fish gut microbiome, as antibiotic-induced dysbiosis directly correlates with reduced viability, impaired immune status, and decreased growth rates in rainbow trout [8].

#### 4.4.1. Pathogen-Induced Changes in the Gut Microbiota of Rainbow Trout

Beyond antibiotic-induced effects, viral infections such as those caused by infectious hematopoietic necrosis virus (IHNV) also induce profound microbiome restructuring. A key feature of IHNV-associated microbiome shifts is their pronounced spatial and temporal heterogeneity, suggesting that no single universal response and necessitating region- and stage-specific analyses. The first evidence of strong site-specificity of the primary response was obtained by Dong et al. [64]: viral invasion induced large-scale taxonomic shifts predominantly in the oral (buccal) mucosa, while the lower gastrointestinal tract remained largely unaffected in the early stages. In the oral mucosa, infection reduced the relative abundance of Pseudomonadota, accompanied by an increase in Cyanobacteria, Bacillota, and representatives of the order Enterobacterales. The loss of dominant parietal commensals was accompanied by a marked accumulation of opportunistic taxa: *Clostridiales*, *Bacteroidiales*, and the genus *Escherichia-Shigella*. In the foregut and distal intestine, alpha diversity indices remained stable, with only a moderate increase in estimated Chao1 richness in the foregut, suggesting regional buffering of dysbiotic disturbances. At the phylum level, healthy fish were dominated by Pseudomonadota and Actinomycetota (mouth, pharynx) or Mycoplasmatota (stomach, intestine). Following infection in the oral cavity, the proportion of Pseudomonadota decreased with a marked increase in Cyanobacteria abundance, a pattern subsequently confirmed in more distal regions under other experimental setups.

However, a complete picture requires consideration of temporal dynamics, which reveal both the mechanisms of pathological shifts and potential pathways for community restoration. This aspect was central to the study by Huang et al. [65], in which metagenomic analysis of the gut microbiota of juvenile trout revealed that alpha diversity indices increased significantly on days 4 and 14 relative to controls but returned to baseline by day 28, indicating a transition from the acute dysbiosis phase to a compensation phase. Beta diversity confirmed this pattern: a clear distinction between infected and control fish on days 4 and 14 was followed by convergence by day 28 in surviving individuals. At the phylum level, Pseudomonadota, Bacillota, Actinomycetota, and Bacteroidota dominated in this study, with the relative abundance of Pseudomonadota decreasing in response to infection (day 4), while Bacillota and Bacteroidota increased markedly. At the order level, Clostridiales, Bacillales, and Bacteroidales increased, whereas Vibrionales and Actinomycetales decreased. The most pronounced increases from days 4 to 14 were observed for Lachnospiraceae, Ruminococcaceae, Bacteroidaceae, and Bacillaceae, whereas Microbacteriaceae decreased. By day 28, the only significant changes remaining were an increase in Moraxellaceae and a decrease in Vibrionaceae, Halomonadaceae, Rickettsiaceae, Pseudomonadaceae, and Streptococcaceae. At the genus level, infection resulted increased both potentially pathogenic bacteria (*Bacteroides*, *Prevotella*, *Alistipes*, *Shigella*) and some beneficial commensals (*Faecalibacterium*, *Bacillus*, *Clostridium*, *Bifidobacterium*), while reducing the abundance of *Halomonas*, *Paracoccus*, *Vibrio*, and *Streptococcus*. Thus, Huang et al. [65] demonstrated the potential for microbiota restoration to the original state in surviving individuals. However, under real aquaculture conditions, this restorative potential may be weakened by additional stressors, raising the question of external modulators of the dysbiotic process.

Temperature is a key modulator, regulating not only viral replication but also shaping dominant bacterial communities and promoting secondary bacterial infections. IHNV infection at low temperatures (12–13 °C) leads to near-complete elimination of the eukaryotic microbiome components (Mucoromycota and Basidiomycota) and a marked decline in Actinomycetota [66]. At the phylum level, low-temperature viral infection stimulates accumulation of Bacillota and Fusobacteriota in the midgut chyme, and at the family and genus level, promotes growth of psychrotolerant opportunists including *Aeromonas cavernicola*, *Pseudomonas stutzeri*, the family Yersiniaceae, and the order Enterobacteriales. In contrast, at higher temperatures (16–17 °C), viral infection results in increased abundance of protective microorganisms of the order Lactobacillales (*Lactococcus*, *Streptococcus* and *Lactococcuslactis*).

A logical extension of this research is whether targeted microbiota modulation via vaccination can enhance infection resistance. Luo et al. [67] examined the effect of an inactivated IHNV vaccine on the intestinal microbiota of triploid trout under various immunization schedules. A comparative analysis of twice-immunized and control groups showed that vaccination induced significant but qualitatively different shifts compared to acute infection. Alpha diversity was significantly lower in immunized fish before infection than in control group. After infection, this indicator remained significantly lower in the vaccinated group than in unimmunized fish, which the authors interpreted as community stabilization rather than classical depletion. Beta diversity confirmed that the impact of IHNV infection on the microbiota of vaccinated fish was significantly less than in unvaccinated fish. At the phylum level, Pseudomonadota and Cyanobacteria dominated in all groups. However, a key difference was that in unvaccinated fish, Cyanobacteria abundance increased markedly after infection and exceeded that of Pseudomonadota, closely replicating the pattern described by Dong et al. [64] for the oral cavity, whereas Pseudomonadota remained dominant in vaccinated fish. At the genus level, vaccinated fish were dominated by *Pseudomonas*, *Gemmobacter*, and *Deefgea*. Before infection, *Pseudomonas* and *Gemmobacter* were significantly more abundant in vaccinated fish, whereas *Deefgea*, *Nannocystis*, and *Flavobacterium* were less abundant than in controls. After infection, the vaccinated group exhibited higher abundance of *Pseudomonas*, *Deefgea*, *Aeromonas*, and *Gemmobacter*, while *Flavobacterium* and *Kluyvera* less abundant. These data suggest that vaccination does not completely prevent dysbiosis but modifies its trajectory, shifting the balance toward resistance-associated taxa (*Pseudomonas*, *Gemmobacter*) while suppressing surges in Cyanobacteria and *Flavobacterium*, shifts that have been correlated with pathogenic outcomes in other studies.

Unlike controlled experimental models with IHNV monoinfection, in commercial aquaculture settings, the microbiota encounters bacterial coinfections and therapeutic interventions that can not only recapitulate but also substantially amplify and perpetuate the dysbiotic shifts described above. This aspect was studied in a group of three-year-old trout naturally coinfected with *Flavobacterium psychrophilum* and *Renibacterium salmoninarum* [68]. Dysbiosis during coinfection is accompanied by a marked decline in Chao1 and Shannon indices and a clear separation of samples into distinct clusters of healthy and diseased individuals. In diseased fish, with dominance of Pseudomonadota and a reduced proportion of Bacillota, Mycoplasmatota expands abnormally. At the genus level, healthy fish were dominated by *Bacillus*, *Serratia*, *Pseudomonas*, *Bacteroides*, and *Cetobacterium*, whereas diseased fish harbored the pathogenic genus *Renibacterium*. In the group with high Mycoplasmatota abundance, this phylum dominated. The main consequence of coinfection is the loss of longitudinal anatomical differentiation along the gastrointestinal tract. In healthy fish, the microbial communities of the stomach and intestine are clearly distinct, whereas in diseased individuals, differences between sections are largely lost. This suggests impairment of longitudinal microbial selection mechanisms and a systemic breakdown of intestinal barrier function.

#### 4.4.2. Antibiotic Effects on Rainbow Trout Microbiota

A notable example of microbiome restructuring following antibiotic use comes from Payne et al. [69], who studied the effect of low-dose oral oxytetracycline (35 mg/kg body weight/day) for 7 days, followed by a 14-day withdrawal period. Analysis of distal intestinal contents showed that the antibiotic induced significant alterations in microbiome composition by the end of treatment. Antibiotic-treated fish showed enrichment of taxa such as *Aeromonas*, *Brevinema*, *Deefgea*, and chloroplast-associated sequences (affiliated with Cyanobacteria) likely derived from feed components, the latter suggesting a breakdown in intestinal colonization resistance. Concurrently, the abundance of *Bacillus* and *Clostridium sensu stricto* decreased. Importantly, following antibiotic withdrawal, the trout microbiome did not stabilize; rather, it continued to change. By day 22 (after 14 days of withdrawal), treated fish exhibited significantly higher OTU richness compared to controls, whereas alpha diversity indices did not differ significantly. Beta diversity confirmed that both time and treatment were statistically significant factors. During withdrawal, the abundance of *Pseudomonas*, *Shewanella*, and *Yersinia* (genera known to include trout-pathogenic species) increased at specific time points. The authors concluded that even a low dose of oxytetracycline induces long-term changes, contributing to an increase in the abundance of opportunistic taxa and feed-associated microorganisms.

A similar pattern of persistent suppression of beneficial commensals and prolonged intestinal dysbiosis has also been documented with other antibacterial agents, such as erythromycin (ERY) or multicomponent antibiotic mixtures containing ampicillin, metronidazole, and ciprofloxacin [70]. A series of experiments assessed both short- and long-term effects. In the first setup, fish received ERY-supplemented feed at three concentrations (0.1, 10, and 1000 μg/g feed) for 7 days, followed by a 1-week withdrawal period. In the second setup (combined exposure), fish were exposed to a high dose of ERY (1000 μg/g) and an antibiotic mixture (100 μg/g of each compound) for 7 days, followed by a 14-day withdrawal period. Pronounced dysbiosis had already developed by day 7, characterized by a decrease in the abundance of dominant taxa (reflected in an increased Shannon index) and a marked reduction in the relative abundance of the obligate lactic acid bacterium *Carnobacterium*, accompanied by an increase in potentially pathogenic *Gallicola*. Notably, even after the withdrawal period, *Carnobacterium* abundance did not recover to baseline levels. This confirms a general pattern: antibiotic therapy causes profound and long-lasting damage to the resident microbiota, compromising fish physiology even after drug clearance.

In addition to the direct effects of antibiotics on the trout gut microbiome, the prophylactic use of antibacterial drugs (florfenicol and erythromycin) was investigated in the context of subsequent infection with *Flavobacterium psychrophilum* [71]. This study design simulates real-world scenarios encountered in aquaculture. Trout administered florfenicol (FFN) or ERY for 10 days and were then infected with *F. psychrophilum*. In healthy untreated fish, the gut microbiome was dominated by Pseudomonadota, Mycoplasmatota (represented primarily by *Mycoplasma*), and Spirochaetota. Antibiotic prophylaxis had already induced significant changes by the time of infection. In the FFN group, *Mycoplasma* abundance declined markedly, even to the point of disappearing, while *Sphingomonas*, *Escherichia-Shigella*, and Sphingomonadaceae increased. In the ERY group, changes were less pronounced but also included a decrease in *Mycoplasma* abundance. After infection, untreated fish exhibited a decline in alpha diversity, and by day 24, *Mycoplasma* regained dominance. In contrast, prophylactically treated groups showed higher alpha diversity by day 24, but this was accompanied by an increase in potentially pathogenic bacteria: *Acinetobacter* and *Pseudomonas* in the FFN group, and *Aeromonas* and *Crenobacter* in the ERY group. Notably, *Mycoplasma*, which disappeared after antibiotic treatment, reappeared by day 24, but baseline levels were not fully restored.

However, in aquaculture practice, the clinical picture is often complicated not only by the infection itself but also by subsequent antibiotic therapy, which can have a profound and, importantly, long-lasting impact, thereby perpetuating dysbiotic changes. A 16-month monitoring study of caged trout following an outbreak of mixed bacterial infection (*Yersinia* sp., *Pseudomonasputida*, *Flavobacterium psychrophilum*) and subsequent enrofloxacin treatment revealed profound consequences in the upper gastrointestinal tract [11]. *Lactobacillus* dominated the stomachs of healthy fish; however, combined exposure to infection and fluoroquinolone resulted in its near-complete and irreversible elimination, which persisted throughout the observation period. In the intestine, therapy triggered a reduction in Bacteroides abundance, accompanied by short-term blooms of opportunistic *Clostridium* and *Cetobacterium*. A key marker of post-infection and antibiotic-induced intestinal sequelae was the progressive increase in Mycoplasmataceae. Virtually absent in native fish, this family showed a marked increase during the acute phase, a brief decline following antibiotic administration, and subsequent unchecked expansion over the following year. The establishment of such a stable state with pronounced mycoplasma dominance alongside decreased alpha diversity illustrates that antibacterial drug use can promote the transition of dysbiosis to a chronic stage.

Taken together, despite differences in experimental design, the studies reviewed reveal similar patterns of dysbiotic alterations in the trout gut microbiome. Both pathogen infection and antibiotic treatment induce profound and often prolonged disruption of the intestinal microbial ecosystem. Common patterns include depletion of obligate commensals (e.g., *Mycoplasma*, *Carnobacterium*, *Lactobacillus*, *Bacillus*, *Clostridium*), overgrowth of opportunistic and feed-associated taxa (e.g., *Aeromonas*, *Pseudomonas*, *Acinetobacter*, *Deefgea*, *Yersinia*, *Shewanella*, *Gallicola*), and impaired colonization resistance with incomplete or prolonged recovery following the removal of the stressor. Viral infections (IHNV) cause pronounced spatial and temporal heterogeneity in microbiome responses, while bacterial coinfections disrupt normal anatomical compartmentalization of the gastrointestinal tract. Antibiotic-induced changes can persist for months, with Mycoplasmataceae often expanding as a long-term dysbiosis marker. Identification of metagenomic indicators—such as a decreased Bacillota/Bacteroidota ratio, elimination of gastrointestinal lactobacilli, and expansion of Mycoplasmataceae—offers significant opportunities for the application of NGS technologies in aquaculture veterinary monitoring and for the development of targeted salmonid health management strategies. Key gaps remain, including the functional consequences of these shifts for host metabolism and immunity, and the potential for microbiome-targeted interventions to restore homeostasis after antibiotic therapy.

## 5. Conclusions

The intestinal microbiome of rainbow trout comprises two functionally and ecologically distinct compartments: the lumen-associated allochthonous (transient) pool and the mucosa-associated autochthonous (resident) pool. A stable phylogenetic core, dominated by Mycoplasmatota, Pseudomonadota, and Bacillota, is consistently detected across diverse rearing conditions. However, this core includes both persistent mucosal symbionts and stable transients derived from feed or water, underscoring the need to distinguish between ecological origin and operational assignment to the core. Among the factors influencing the trout gut microbiome, abiotic stressors (elevated temperature) and therapeutic interventions (antibiotics) trigger the most profound and potentially irreversible alterations, often overriding beneficial dietary effects. Pathogenic infections can exacerbate these disruptions, particularly compromising mucosal barrier function.

Despite recent advances, several knowledge gaps remain. First, the ecological origin and functional roles of many core taxa are still poorly understood, particularly whether they are true symbionts or environmental contaminants. Second, methodological heterogeneity across studies, including differences in sampling (mucus vs. contents), DNA extraction, and sequencing platforms, hampers direct comparisons and limits the generalizability of findings. Third, the functional consequences of microbiota shifts for host health and metabolism remain largely unexplored, with most studies limited to taxonomic inventories.

Methodological considerations are critical for interpreting metagenomic data. The transition from short-read to long-read 16S rRNA sequencing enables higher taxonomic resolution, revealing that some previously assumed core taxa may be methodological artifacts. This highlights the need for standardization of sampling protocols, DNA extraction methods, and primer selection to ensure comparability across studies.

Addressing these gaps requires standardization of protocols, establishment of baseline reference ranges for a healthy microbiome, and integration of functional metagenomics with host physiological parameters in long-term field studies. Such approaches will move beyond descriptive taxonomy toward a mechanistic understanding of host–microbe interactions under real-world aquaculture conditions.

The observed dynamics of the gut metagenome provide a framework for defining a reference range for a healthy microbiome in rainbow trout. Such baseline data would enable early dysbiosis diagnosis and support the development of sustainable feed formulations that maintain intestinal homeostasis. Future research should prioritize long-term field studies integrating microbiome profiling with host physiological parameters and employing metagenomic functional analysis to move beyond taxonomic inventories toward understanding host–microbe interactions under real-world aquaculture conditions.

## Figures and Tables

**Table 1:** Core bacterial taxa in the gut microbiome of rainbow trout *O. mykiss*. The table summarizes taxa reported in the reviewed studies, including dominant and minor/transient core members, and distinguishes between luminal, mucosal, and whole-intestine (combined) datasets.

Compartment	Phylum (Former Name)	Families	Genera	Species	Reference(s)
**Intestinal lumen**	Bacillota (Firmicutes)	Bacillaceae, Carnobacteriaceae, Clostridiaceae, Enterococcaceae, Lactobacillaceae, Leuconostocaceae, Staphylococcaceae, Streptococcaceae	*Carnobacterium*, *Clostridium*, *Lactobacillus*, *Lactococcus*, *Streptococcus*	*Clostridium perfringens*, *C. gasigenes*	[3,8,12]
	Pseudomonadota (Proteobacteria)	Aeromonadaceae, Campylobacteraceae, Comamonadaceae, Enterobacteriaceae, Moraxellaceae, Oxalobacteraceae, Pasteurellaceae, Pseudomonadaceae, Rhodobacteraceae, Shewanellaceae, Sphingomonadaceae, Vibrionaceae	*Acinetobacter*, *Aeromonas*, *Buttiauxella*, *Escherichia-Shigella*, *Erwinia*, *Hafnia*, *Pantoea*, *Plesiomonas*, *Proteus*, *Pseudomonas*, *Rahnella*, *Ralstonia*, *Serratia*, *Yersinia*	*Enterobacter intermedius*; *Erwinia paradisiaca*; *Rahnella aquatilis*, *R. nicotianae*; *Serratia entomophila*, *S. proteamaculans*; *Yersinia enterocolitica*, *Y. ruckeri*	[3,8,12]
	Bacteroidota (Bacteroidetes)	Bacteriodaceae, Flavobacteriaceae, Sphingobacteriaceae	*Capnocytophaga*, *Flavobacterium*, *Prevotella*	*Flavobacterium columnare*, *F. psychrophilum*	[3,8]
	Fusobacteriota	Fusobacteriaceae	*Cetobacterium*, *Fusobacterium*	*Cetobacterium somerae*; *Fusobacterium nucleatum* subsp. *nucleatum*	[3,8]
	Actinomycetota (Actinobacteria)	Corynebacteriaceae, Microbacteriaceae, Micrococcaceae, Nocardiaceae, Propionibacteriaceae	Not specified	Not specified	[8]
	Mycoplasmatota (Tenericutes)	Mycoplasmataceae	*Mycoplasma*	Not specified	[8,12]
	Spirochaetes	Not specified	Not specified	Not specified	[8]
	Cyanobacteria	Not specified	Not specified	Not specified	[8]
**Mucosal layer**	Bacillota (Firmicutes)	Bacillaceae, Carnobacteriaceae, Clostridiaceae, Enterococcaceae, Lactobacillaceae, Leuconostocaceae, Peptostreptococcaceae, Staphylococcaceae, Streptococcaceae	*Acetanaerobacterium*, *Bacillus*, *Carnobacterium*, *Catellicoccus*, *Clostridium*, *Enterococcus*, *Lactobacillus*, *Lactococcus*, *Leuconostoc*, *Streptococcus*, *Weissella*	*Bacillus nitratireducens*; *Carnobacterium maltaromaticum*; *Clostridium bifermentans*, *C. carnis*, *C. gasigenes*, *C. tertium*; *Intestinibacter bartlettii*; *Lactobacillus fuchuensis*; *Lactococcus cremoris*, *L. garvieae*; *Latilactobacillus sakei*; *Leuconostoc gasicomitatum*; *Romboutsia lituseburensis*; *Weissella koreensis*	[8,12]
	Pseudomonadota (Proteobacteria)	Aeromonadaceae, Campylobacteraceae, Chromobacteriaceae, Comamonadaceae, Desulfovibrionaceae, Enterobacteriaceae, Moraxellaceae, Oceanospirillaceae, Oxalobacteraceae, Pseudomonadaceae, Rhodobacteraceae, Sphingomonadaceae, Xanthomonadaceae	*Acinetobacter*, *Aeromonas*, *Deefgea*, *Enterobacter*, *Escherichia*, *Maricurvus*, *Moritella*, *Neptuniibacter*, *Pantoea*, *Photobacterium*, *Pseudomonas*, *Psychrobacter*, *Ralstonia*, *Shigella*	*Acinetobacter harbinensis*, *A. lwoffii*, *A. schindleri*, *A. boissieri*; *Aeromonas hydrophila* subsp. *dhakensis*, *A. hydrophila* subsp. *hydrophila*, *A. sanarellii*, *A. sobria*; *Deefgea chitinilytica*, *D. piscis*, *D. rivuli*, *D. salmonis*; *Enterobacter intermedius*; *E. coli*; *Iodobacter fluviatilis*; *Neptuniibacter caesariensis*, *N. halophilus*; *Oceanospirillum linum*; *Pseudomonas antarctica*, *P. fragi*, *P. meridiana*, *P. yamanorum*; *Psychrobacter aquimaris*, *P. cibarius*, *P. frigidicola*, *P. fulvigenes*, *P. glacincola*; *Ralstonia insidiosa*, *R. nicotianae*, *R. pickettii*, *R. solanacearum*; *Shigella sonnei*	[3,5,8,12]
	Bacteroidota (Bacteroidetes)	Bacteriodaceae, Flavobacteriaceae, Sphingobacteriaceae	*Flavobacterium*	*Flavobacterium algicola*, *F. antarctium*, *F. degerlachei*, *F. frigidarium*	[3,5,8]
	Fusobacteriota	Fusobacteriaceae	*Cetobacterium*	*Cetobacterium ceti*, *C. somerae*; *Fusobacterium perfoetens*	[8]
	Actinomycetota (Actinobacteria)	Corynebacteriaceae, Micrococcaceae, Propionibacteriaceae	*Rhodococcus*	*Rhodococcus qingshengii*	[8,12]
	Mycoplasmatota (Tenericutes)	Mycoplasmataceae	*Mycoplasma*, *Malacoplasma*, *Mesomycoplasma*	*Candidatus Mycoplasma salmoninae mykiss*, *Mycoplasma iowae*; *Malacoplasma muris*; *Mesomycoplasma moatsii*,	[3,5,8,12]
	Spirochaetes	Brevinemataceae	Not specified	Not specified	[8]
	Planctomycetes	Not specified	Not specified	Not specified	[8]
**Whole intestine**	Bacillota (Firmicutes)	Bacillaceae, Carnobacteriaceae, Clostridiaceae, Enterococcaceae, Lactobacillaceae, Leuconostocaceae, Staphylococcaceae, Streptococcaceae	*Acetanaerobacterium*, *Bacillus*, *Catellicoccus*, *Enterococcus*, *Lactobacillus*, *Lactococcus*, *Leuconostoc*, *Streptococcus*, *Weissella*	Not specified	[4,8]
	Pseudomonadota (Proteobacteria)	Aeromonadaceae, Enterobacteriaceae, Moraxellaceae, Oxalobacteraceae, Pseudomonadaceae, Vibrionaceae, Xanthomonadaceae	*Acinetobacter*, *Maricurvus*, *Moritella*, *Pantoea*, *Photobacterium*, *Pseudomonas*	Not specified	[4,8]
	Bacteroidota (Bacteroidetes)	Bacteroidaceae, Flavobacteriaceae	Not specified	Not specified	[4,8]
	Fusobacteriota	Fusobacteriaceae	Not specified	Not specified	[4,8]
	Actinomycetota (Actinobacteria)	Corynebacteriaceae	Not specified	Not specified	[4,8]
	Mycoplasmatota (Tenericutes)	Mycoplasmataceae	*Mycoplasma*	Not specified	[4,8]
	Spirochaetes	Brevinemataceae	*Brevinema*	Not specified	[4,8]
	Deinococcota	Deinococcaceae	Not specified	Not specified	[4]
	Thermodesulfobacteria	Not specified	Not specified	Not specified	[4]
	Verrucomicrobiota	Opitutae	Not specified	Not specified	[4]

**Table 2:** Summary of exogenous and endogenous factors modulating the gut microbial community in rainbow trout *O. mykiss*. ↑ indicates increase in relative abundance or diversity; ↓ indicates significant decrease; FM—fish meal.

Factor	Specific Trigger	Key Taxonomic Shifts (↑ Increase/↓ Decrease)	Reference(s)
**Dietary modulation**			
**Replacement of FM with plant-based ingredients**	Soybean, pea, rapeseed, lupine, wheat	↑ Bacillota/Pseudomonadota ratio; ↑ *Streptococcus*, ↑ *Leuconostoc*, ↑ *Weissella*; ↓ Pseudomonadota; ↑ Firmicutes, ↓ Proteobacteria	[13,14,15,16,17]
**Insect meal**	*Hermetia illucens*	↑ Bacillota; ↓ Pseudomonadota; ↑ Lactobacillus, ↑ *Bacillus*, ↑ *Carnobacterium*, ↑ *Oceanobacillus*, ↑ *Paenibacillus*; ↓ *Aeromonas*; ↓ Mycoplasmoidaceae; ↓ *Peptostreptococcus*	[18,19,20,21]
	*Tenebrio molitor*	No adverse effects; moderate community shifts; ↓ Proteobacteria	[22,23]
	*Gryllodes sigillatus*	Reduced alpha diversity, ↓ Mycoplasmoidaceae, ↑ Streptococcaceae; ↓ *Peptostreptococcus*	[21]
**Probiotic**	*Pediococcus acidilactici*	Stabilization of microbiota during *Yersinia ruckeri* infection ↓ *Mycoplasma*; ↑ Proteobacteria, ↑ Clostridiales, ↑ Enterobacteriaceae, ↑ *Pseudomonas*, ↑ *Massilia*, ↑ Weissella, ↑ *Staphylococcus*	[24,25]
	Multispecies formulations (*Bacillus* sp., *Pediococcus* sp., *Enterococcus* sp., *Lactobacillus* sp.)	Increase in OUTs and Shannon index	[26]
	*Bacillus velezensis*, *Lactobacillus sakei*	*B. velezensis*: ↑ Lachnospiraceae, ↑ Ruminococcus, ↑ *Bacillus coagulans*, ↑ *Leptotrichia*, ↑ *Bacillus coagulans*, ↑ Porphyromonadaceae, ↑ *Anaerococcus*, ↑ *Photobacterium*; *L. sakei*: ↑ Paenibacillaceae, ↑ *Eubacterium hallii* ↓ pathogenic taxa	[27]
	*Carnobacterium maltaromaticum*	↓ *Pseudomonas fluorescens*, ↓ *Aeromonas hydrophila*, ↓ *Staphylococcus* spp., ↓ *Clostridium* spp.	[28]
	*Bacillus cereus var. Toyoi*	More stable composition (less inter-individual variability)	[29]
	*Weissella confusa*	Increased levels of lactic acid bacteria and total bacteria count	[30]
**Prebiotic**	Mannan oligosaccharides	↓ *Aeromonas*, ↓ *Vibrio*; ↑ Bacillota, ↑ Fusobacteria ↓ *Micrococcus* spp., ↑ *Enterococcus* spp. and Enterobacariaceae, ↑ *Pseudomonas* spp., ↓ *Escherichia-Shigella*	
	Inulin	Modulation of chyme microbiota only (not parietal); ↑ *Weissella*, ↑ *Streptococcus*	[31,32]
	β-glucan (*Saccharomyces cerevisiae*)	↑ Actinobacteria (*Aurantimicrobium*); ↓ Bacillota (*Carnobacterium*, *Deefgea*)	[33]
	Arabinoxylan (10% high concentration)	↑ Firmicutes/Bacteroidetes ratio; ↑ opportunistic *Stenotrophomonas*	[34]
**Soluble non-starch polysaccharides**		↑ *Pseudomonas aeruginosa*, ↑ *Photobacterium kishitanii*	[35]
**Postbiotics**		↑ Tenericutes/Fusobacteria ratio; ↓ sulfate-reducing *Desulfovibrio*	[36,37]
**Plant extracts**	Capsaicin (*Capsicum* spp.)	↑ Clostridiaceae; affects rare taxa	[38,39]
	Garlic, Chinese yam, Ganoderma lucidum) + lentinan (shiitake)	Suppression of pathogenic *Mycobacterium*, *Nannocystis*; restoration of homeostasis after IHNV infection	[39,40]
**Mushroom stipes**	*Agaricus bisporus*, *Lentinula edodes*, *Pleurotus ostreatus*	↓ Desulfobacterota, ↓ *Staphylococcus*; ↑ beneficial taxa	[41]
**Organic acids + essential oils mixtures**		↓ *Aeromonas hydrophila*, ↓ *Acinetobacter*; ↑ *Streptococcus*, ↑ *Fusobacterium*	[42,43,44]
**Sodium butyrate**		↑ species richness; Firmicutes → Proteobacteria shift; *Mycoplasma* → *Aeromonas* dominance shift; ↓ opportunistic Proteobacteria	[45,46]
**Copper(I) complexes**		↑ *Pseudomonas*, ↑ *Corynebacterium*	[47]
**Glutathione (400 mg/kg)**		↑ alpha diversity; ↓ *Arcobacter*	[48]
**Nano-selenium (nano-Se, 5 mg/kg) + acute heat stress (24 °C)**		Heat stress: ↓ *Ralstonia*, ↓ Pseudomonadota; ↑ Actinomycetota, ↑ Bacillota; ↑ stress biomarkers (*Methylobacterium*, *Akkermansia*, *Deinococcus*); nano-Se restores baseline profiles	[49]
**Combined dietary transition (FM → plant-based) + stocking density (low vs. high)**		Stable core: Bacilli, Clostridia, Alphaproteobacteria, Gammaproteobacteria, Betaproteobacteria; Plant-based + low-density: ↑ *Bacillus* spp.; FM + high density: ↑ *Clostridium*; plant-based: ↑ *Lactobacillus*, ↑ *Streptococcus*; synergistic effect on *Staphylococcus*	[50]
**Rearing system/Biotope**			
**Wild and farmed trout**		Wild: ↑ Bacillota, ↑ Fusobacteriota, ↑ Cyanobacteria, ↑ Pseudomonadota, ↑ Bacteroidota; ↑ Cetobacterium, ↑ *Clostridium sensu stricto*; absence of *Mycoplasma*, *Pseudomonas*, *Weissella* (core for farmed)	[51]
**Open-air lake farms and controlled laboratory aquaria**		Farmed: ↑ *Photobacterium*, ↑ *Catellicoccus*, ↑ *Moritella*, ↑ *Ureibacillus*, ↑ *Paralactobacillus*, ↑ *Psychrilyobacter*, ↑ *Thermobacillus*, ↑ *Lactobacillus*, ↑ *Fusobacterium*; Aquarium: ↑ *Sphaerotilus*, ↑ *Maricurvus*, ↑ *Weissella*	[4]
**RAS vs. flow-through**		Flow-through: ↑ *Lactobacillus*, ↑ *Lactococcus*, ↑ *Clostridium*, ↑ *Catellicoccus*, ↑ *Fusobacterium*, ↑ *Ureibacillus*, ↑ *Paralactobacillus*, ↑ *Thermobacillus*; RAS/Aquaria: ↑ Gammaproteobacteria (*Aeromonas*, *Lelliottia*, *Maricurvus*), ↑ *Weissella*, ↑ *Enterococcus*, ↑ *Streptococcus*; distal intestine: *Mycoplasma* predominates	[4,52,53]
**Hindgut isolation from other organs (RAS)**		Distal intestine: ↑ *Mycoplasma*; exclusion of strict aerobes (*Flavobacterium*, *Crocinitomix*); facultative/obligate anaerobes dominate	[53]
**Farm-specific and seasonal variation**		Dominance of Enterobacteriaceae OR mixture of *Carnobacterium*, *Pseudomonas*, *Shewanella*, *Acinetobacter*, *Plesiomonas* depending on farm and season; resident taxa: *Carnobacterium piscicola*, *Clostridium botulinum*, uncultured coccoid bacteria	[54]
**Stress**			
**Acute stress**	Water level drop + mechanical pursuit	Disappearance of *Acinetobacter*, *Rhodococcus*; ↑ *Pseudomonas dominance*; appearance of *Arthrobacter*, *Microbacterium*, *Micrococcus* in feces	[55]
	Fasted fish (3 days)	Parietal mucus buffering capacity more resistant; reduced epithelial detachment	[55]
**Chronic stress**	Repeated handling—stress-susceptible genotype	↑ alpha diversity in intestinal contents (regardless of diet); beta diversity strongly influenced by stress	[56]
	Stress-resistant genotype	Alpha diversity stable; complex synergistic interaction between stress and diet (beta diversity)	[56]
	Phylum-level marker	↑ Fusobacteriota; ↑ *Cetobacterium* (key marker); FM-based diet + stress: ↑ *Cetobacterium*, ↑ *Photobacterium*, ↑ *Plesiomonas*; Plant-based diet + stress: ↑ *Bifidobacterium*, ↑ *Candidatus Microthrix*	[56]
	Mucosal vs. luminal partitioning	Contents: ↑ *Bifidobacterium*, ↑ *Staphylococcus*, ↑ *Corynebacterium*, ↑ Bacteroides; Mucosa: *Mycoplasma*, *Cetobacterium*, *Photobacterium*, *Brevinema* dominate	[57]
**Stocking density**	Excessively low (12 kg/m^3^)	↑ opportunistic pathogens: *Pseudomonas putida*, *Acinetobacter lwoffii*, *Pseudomonas alcaligenes*, *Shewanella* spp.	[58]
	Moderate (43 kg/m^3^)	↑ immunomodulatory commensals: *Cetobacterium somerae*, *Romboutsia lituseburensis*, *Lactobacillus plantarum*	[58]
**Starvation/severe feed restriction**		↓ *Bifidobacterium*; ↑ opportunistic *Helicobacter*, ↑ *Staphylococcus*, ↑ *Pseudomonas* (reversible within 7–14 days)	[59]
**Water temperature**	Upper thermal optimum (18 °C)	FM diet: pronounced diversity decline; yeast supplementation: partial protective effect; 18 °C + yeast: ↑ Mycoplasmatales; 11 °C + FM: ↑ Lactobacillales (↑ Leuconostocaceae, ↑ *Lactobacillus reuteri*, ↑ *Photobacterium*); 18 °C: ↑ Aeromonaceae (chyme); parietal microbiome stable	[60]
	Temperature shift (14 °C → 18 °C)—dominates over diet	14 °C: autochthonous Mycoplasmataceae dominate; 18 °C: replacement by opportunistic Aeromonadaceae and Enterobacteriaceae (key heat stress indicators)	[61]
	Acute heat stress (22.5–24.5 °C, 24 h)	↑ Mycoplasmatota, ↑ Bacillota; ↑ *Mycoplasma*, ↑ *Cetobacterium*, ↑ *Aeromonas*, ↑ *Shewanella*, ↑ *Clostridium*; ↓ *Lactobacillus* spp., ↓ *Coldibacterium*, ↓ *Morganella*, ↓ *Enterobacter*, ↓ *Lawsonia*; Biomarkers: 16 °C—*Cloacibacterium normanense*, Prevotellaceae, *Microbacterium*, *Morganella*, *Lactobacillus fermentum*; 22.5 °C—*Mycoplasma* spp., Mycoplasmataceae, Tenericutes; 23.5 °C—Bacilli; 24.5 °C—Betaproteobacteria	[35]
	Prolonged heat stress (24 °C, 21 days)—mucosal niche	↓ *Mycoplasma*; ↑ Enterobacteriaceae (*Escherichia-Shigella*, *Klebsiella*); ↑ *Aeromonas veronii*, ↑ *Aeromonas hydrophila*	[62,63]
	Prolonged heat stress (24 °C, 21 days)—chyme	↓ Bacillota, ↓ Bacteroidota; ↑ Pseudomonadota; ↓ *Bacillus*, ↓ *Clostridium butyricum*, ↓ *Acinetobacter johnsonii*, ↓ *A. lwoffii*; ↑ uncultured Escherichieae	[62,63]
**Pathogens**			
**IHNV infection**	Foregut/distal intestine (early stage)	Alpha diversity stable; moderate ↑ Chao1 in foregut; regional buffering of dysbiosis	[64]
	Temporal dynamics (days 4, 14, 28)	Day 4: ↓ Pseudomonadota; ↑ Bacillota, ↑ Bacteroidota; ↑ Clostridiales, ↑ Bacillales, ↑ Bacteroidales; ↓ Vibrionales, ↓ Actinomycetales; Day 4–14: ↑ Lachnospiraceae, ↑ Ruminococcaceae, ↑ Bacteroidaceae, ↑ Bacillaceae; ↓ Microbacteriaceae; Day 28: ↑ Moraxellaceae; ↓ Vibrionaceae, Halomonadaceae, Rickettsiaceae, Pseudomonadaceae, Streptococcaceae; ↑ *Bacteroides*, ↑ *Prevotella*, ↑ *Alistipes*, ↑ *Shigella*, ↑ *Faecalibacterium*, ↑ *Bacillus*, ↑ *Clostridium*, ↑ *Bifidobacterium*; ↓ *Halomonas*, ↓ *Paracoccus*, ↓ *Vibrio*, ↓ *Streptococcus*	[65]
	IHNV infection—low temperature (12–13 °C)	↑ Bacillota, ↑ Fusobacteriota (midgut chyme); ↑ psychrotolerant opportunists: *Aeromonas cavernicola*, *Pseudomonas stutzeri*, Yersiniaceae, Enterobacteriales; elimination of Mucoromycota, Basidiomycota; ↓ Actinomycetota	[66]
	IHNV infection—high temperature (16–17 °C)	↑ protective Lactobacillales (*Lactococcus*, *Streptococcus*, *Lactococcus lactis*)	[66]
	Inactivated IHNV vaccine (twice-immunized)	↓ Cyanobacteria surge; Pseudomonadota remain dominant; ↑ *Pseudomonas*, ↑ *Gemmobacter*, ↑ *Deefgea*; ↓ *Flavobacterium*, ↓ *Nannocystis*	[67]
**Coinfection**	*Flavobacterium psychrophilum* + *Renibacterium salmoninarum*	Healthy: ↑ *Bacillus*, ↑ *Serratia*, ↑ *Pseudomonas*, ↑ *Bacteroides*, ↑ *Cetobacterium*; Diseased: ↑ pathogenic *Renibacterium*; ↑ *Pseudomonadota*; ↓ Bacillota; ↑ Mycoplasmatota, loss of longitudinal anatomical differentiation (stomach vs. intestine)	[68]
**Antibiotics**			
**Antibiotic**	Oral oxytetracycline (35 mg/kg/day, 7 days + 14-day withdrawal)	↑ *Aeromonas*, ↑ *Brevinema*, ↑ *Deefgea*, ↑ chloroplast-associated sequences (Cyanobacteria from feed); ↓ *Bacillus*, ↓ *Clostridium sensu stricto*; withdrawal: ↑ *Pseudomonas*, ↑ *Shewanella*, ↑ *Yersinia*	[69]
	Erythromycin—7 days + withdrawal	↓ dominant taxa; ↓ obligate lactic acid bacterium *Carnobacterium* (no recovery after withdrawal); ↑ potentially pathogenic *Gallicola*	[70]
	Antibiotic mixture (ampicillin, metronidazole, ciprofloxacin)—7 days + withdrawal	↓ *Carnobacterium* (no recovery); ↑ potentially pathogenic *Gallicola*	[70]
**Antibiotic and bacterial infection**	Florfenicol + *F. psychrophilum infection*	↓ *Mycoplasma* (disappearance); ↑ *Sphingomonas*, ↑ *Escherichia-Shigella*, ↑ Sphingomonadaceae; Post-infection: ↑ *Acinetobacter*, ↑ *Pseudomonas*; *Mycoplasma* reappears by day 24 but not fully restored	[71]
	Erythromycin + *F. psychrophilum infection*	↓ *Mycoplasma* (less pronounced than FFN); Post-infection: ↑ *Aeromonas*, ↑ *Crenobacter*; *Mycoplasma* reappears by day 24 but not fully restored	[71]
	Mixed infection + enrofloxacin—intestine (16-month monitoring)	↓ Bacteroides; short-term blooms: ↑ *Clostridium*, ↑ *Cetobacterium*; progressive increase in Mycoplasmataceae	[11]

## Data Availability

No new data were created or analyzed in this study. Data sharing is not applicable to this article.

## Abbreviations

The following abbreviations are used in this manuscript:

GIT	Gastrointestinal tract
OTU	Operational Taxonomic Unit
NGS	Next-Generation Sequencing
RAS	Recirculating aquaculture systems
ERY	Erythromycin
IHNV	Infectious hematopoietic necrosis virus
FFN	Florfenicol
